# Endoscopic ultrasonography-guided choledochoduodenostomy without tract dilation using a novel ultra-tapered slim-delivery metallic stent.

**DOI:** 10.1055/a-2607-8148

**Published:** 2025-06-18

**Authors:** Haruo Miwa, Ritsuko Oishi, Shotaro Tsunoda, Kazuki Endo, Yuichi Suzuki, Hiromi Tsuchiya, Shin Maeda

**Affiliations:** 1 26437 Gastroenterological Center, Yokohama City University Medical Center, Yokohama, Japan; 2 Gastroenterology, Yokohama City University Graduate School of Medicine, Yokohama, Japan

Several techniques for endoscopic ultrasonography-guided choledochoduodenostomy (EUS-CDS) without tract dilation have been reported to reduce the risk of bile leakage 1 2 3 4 . However, bile leakage may still occur, particularly when the insertion of the stent delivery system is challenging. A novel self-expandable metallic stent (SEMS) with a 7-Fr slim delivery system (Niti-S EUS-BD system End Bare Single Flare; Taewoong Medical Co., Ltd., Gimpo, South Korea) features an ultra-tapered tip designed for use with a 0.025-in guidewire ( Fig. 1 ). Its cross-wire structure provides a high radial force that minimizes the risk of stent migration. Herein, we present a case of EUS-CDS performed without tract dilation using this novel SEMS ( Video 1 ).

**Fig. 1:**
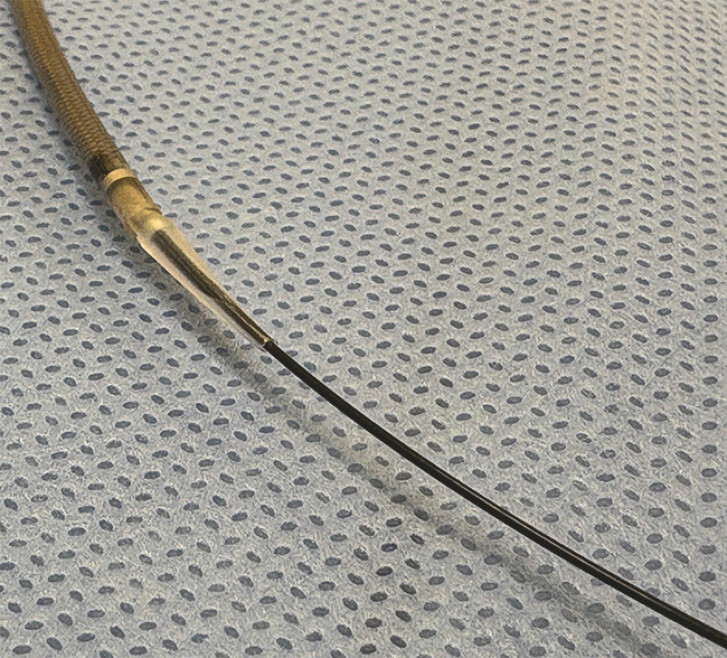
A novel self-expandable metallic stent with a 7-Fr slim delivery system (Niti-S EUS-BD system End Bare Single Flare; Taewoong Medical Co., Ltd., Gimpo, South Korea) featuring an ultra-tapered tip that minimizes the gap with a 0.025-in guidewire.

A novel ultra-tapered slim-delivery metallic stent was used to perform endoscopic ultrasonography-guided choledochoduodenostomy without the need for tract dilation in a 64-year-old woman with unresectable pancreatic cancer, biliary obstruction, and liver metastasis.Video 1

A 64-year-old woman with unresectable pancreatic cancer complicated by biliary obstruction was admitted to our hospital. Computed tomography revealed multiple metastases in the left liver lobe ( Fig. 2 ). Initial transpapillary biliary drainage was attempted, but the guidewire could not be passed through the stricture. EUS-CDS was subsequently performed. A dilated common bile duct was punctured using a 19-gauge Franseen needle (TopGain; Medi-Globe GmbH, Grassau, Germany), and a 0.035-in guidewire was introduced. An attempt was made to insert a fully covered SEMS with an 8-Fr delivery system, but the tip of the system was unable to pass through the bile duct wall.

**Fig. 2:**
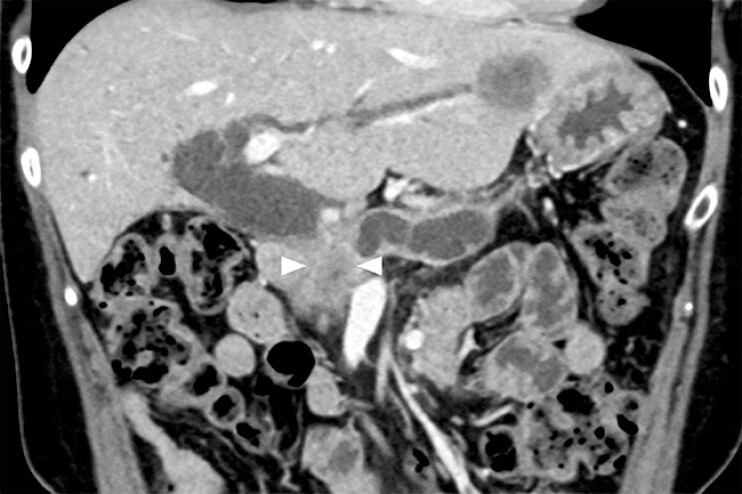
Computed tomography of a 64-year-old woman with unresectable pancreatic cancer and biliary obstruction (arrowheads) revealed liver metastasis in the left lobe.

A tapered catheter was then inserted, and the guidewire was exchanged for a 0.025-in version (VisiGlide 2; Olympus Medical Systems, Tokyo, Japan). Utilizing the ultra-tapered tip and the 7-Fr slim delivery system, the novel SEMS (8-mm diameter, 10-cm length) was advanced smoothly into the bile duct without the need for tract dilation. The stent was successfully deployed from the left hepatic duct to the duodenum ( Fig. 3 ). The patient recovered uneventfully and was discharged without adverse events.

**Fig. 3:**
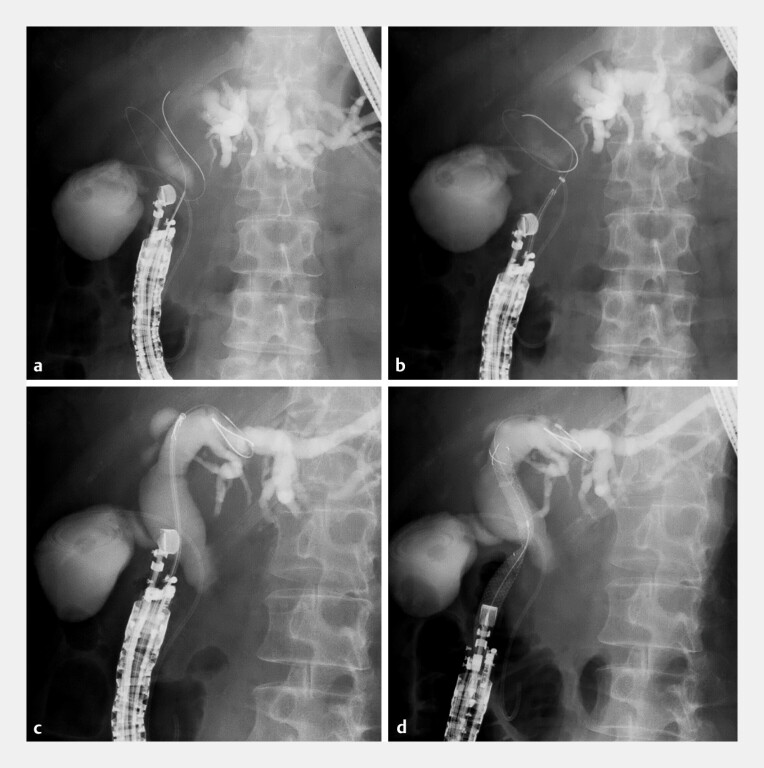
Endoscopic ultrasonography-guided choledochoduodenostomy. **a** The dilated common bile duct was punctured using a 19-gauge Franseen needle. **b** A fully covered metallic stent with an 8-Fr delivery system could not pass through the bile duct wall. **c** The novel metallic stent with an ultra-tapered tip and 7-Fr slim-delivery system was smoothly advanced into the bile duct. **d** The metallic stent was placed successfully.

To the best of our knowledge, this is the first reported case of EUS-CDS performed without tract dilation using a novel SEMS with an ultra-tapered slim delivery system. This simple approach offers a safe technique for EUS-CDS.

Endoscopy_UCTN_Code_TTT_1AS_2AD

## Notes

Endoscopy E-Videos https://eref.thieme.de/e-videos
*E-Videos* is an open access online section of the journal *Endoscopy* , reporting on interesting cases and new techniques in gastroenterological endoscopy. All papers include a high-quality video and are published with a Creative Commons CC-BY license. Endoscopy E-Videos qualify for HINARI discounts and waivers and eligibility is automatically checked during the submission process. We grant 100% waivers to articles whose corresponding authors are based in Group A countries and 50% waivers to those who are based in Group B countries as classified by Research4Life (see: https://www.research4life.org/access/eligibility/ ). This section has its own submission website at https://mc.manuscriptcentral.com/e-videos .
